# The influence of menopause symptoms on workplace mental health among Irish women: A preliminary study

**DOI:** 10.1016/j.cpnec.2025.100324

**Published:** 2025-10-22

**Authors:** Wendyrose Smith, Sarah M. Cooney, Akansha M. Naraindas

**Affiliations:** a Department of Psychology, Dublin Business School, Dublin, Republic of Ireland; b School of Psychology, University College Dublin, Dublin, Republic of Ireland; c Centre for Public Health, Queens University Belfast, Belfast, UK

**Keywords:** Menopause, Women's health, Work ability, Workplace confidence, Workplace mental health, Organisational support

## Abstract

**Introduction:**

Women's experiences of menopause are shaped not only by biological changes but also by the social contexts in which they occur, such as the workplace. This study examines how psychological, somatic and urogenital menopause symptoms affect occupational self-efficacy, work ability, perceived health and work quality, and perceived workplace support among Irish women.

**Methods:**

A total of 121 participants (Mage = 49.9, SD = 4.9, Range = 37–62) experiencing perimenopausal or postmenopausal symptoms participated in an online survey. Participants completed validated measures assessing menopause symptom severity, occupational self-efficacy, work ability, health and work quality, perceived workplace support, and menopause-related absenteeism.

**Results:**

Psychological symptoms were significantly associated with lower occupational self-efficacy and work ability, whilst somatic and urogenital symptoms were not. Higher psychological, somatic and urogenital symptoms were correlated with multiple health and work quality measures. Organisational support was significantly associated with lower symptom severity. No significant relationships were found between symptom severity and absenteeism or reduced working hours.

**Conclusion:**

Psychological menopause symptoms impact key workplace outcomes, while organisational support appears protective. These findings underscore the importance of menopause-informed policies in promoting the well-being, confidence, and retention of the workforce.

## Highlights

• Menopause symptoms can affect mental health within workplace social and structural contexts.
• Psychological menopause symptoms predict lower work ability and self-efficacy.
• Psychological, Somatic and urogenital symptoms are linked to health and work quality outcomes.
• Organisational support is associated with lower overall symptom severity.
• Results highlight the need for menopause-informed workplace mental health policies.

## 1 Introduction

Menopause represents a major life transition with physiological, psychological, and social implications. Despite this, menopause remains underrepresented in occupational health research and policy. This oversight is especially concerning given the critical role that employment plays in supporting women's mental health [1]. Menopause is the permanent cessation of menstruation due to loss of ovarian follicular activity, diagnosed retrospectively after 12 consecutive months without menstruation [47]. Perimenopause is the transitional period spanning the years before the final menstrual period and the 12 months after it; onset, duration, and impact vary widely but typically last 5–10 years [47]. Symptoms are multisystem, with vasomotor symptoms (hot flushes/night sweats) being the most characteristic of the perimenopause and are increasingly recognised as a potential preclinical marker [48]. Additionally, urogenital changes (e.g., vaginal dryness, urinary symptoms), psychological symptoms (e.g., elevated risk of depression and anxiety), and cognitive complaints (e.g., “brain fog”) are frequently reported [49]. Symptoms vary widely in type and severity across individuals and populations worldwide, with potential for substantial disruption to daily functioning, including workplace performance and wellbeing [3].

In 2024, Ireland's female labour force participation reached a record high of 61.4 %, with the highest employment rates among women aged 35–44 [3]. Many in this group are likely to experience menopausal symptoms while managing demanding roles, yet the workplace impacts of these symptoms remain under-researched. Recent data suggests that among 4014 UK women surveyed, nearly two-thirds reported that menopause symptoms had negatively affected them at work [4]. Approximately 10 % left their jobs, 14 % reduced their hours, and 8 % turned down promotions. The most common challenges cited were those related to psychological symptoms, specifically reduced concentration and increased stress, symptoms that may interfere with performance, confidence, and career progression. Compared with premenopausal women, those in the menopausal transition report a broader burden of psychological symptoms—memory and concentration difficulties, depression, anxiety, insomnia, fatigue, irritability, and heightened distress—that could adversely affect quality of life in the workplace [6]. Despite this, the evidence base remains limited: most studies prioritise vasomotor and other physical symptoms or global quality-of-life indices, often in clinical rather than occupational samples [7].

Occupational self-efficacy, defined as an individual's belief in their ability to perform job tasks successfully [8], is another well-established predictor of workplace motivation, persistence, and performance [9]. During menopause, psychological symptoms such as changes in memory, attention, and emotional stability can undermine self-efficacy, erode confidence, and discourage women from pursuing career advancement or maintaining demanding roles [10]. Although direct studies linking menopause to occupational self-efficacy specifically are limited, related research suggests that symptom burden can affect perceived competence and lead to elevated workplace anxiety [6]. Menopausal symptoms also impact work ability (i.e. the fit between job demands and an employee's capacity to fulfil them [12]). Somatic and psychological symptoms like insomnia, chronic pain, and emotional instability may reduce physical stamina, leading to lower perceived work capacity and greater occupational strain [13]. To date, only one study from the Netherlands has examined work ability in menopausal women, reporting that those with severe symptoms had significantly lower work ability and higher absenteeism compared to asymptomatic controls [14]. However, the study assessed overall symptom severity and did not explore specific symptom groups, leaving it unclear how particular symptoms impact work ability.

Beyond self-perception of performance, menopause can also affect perceptions of health and work quality [15]. Greater severity of menopausal symptoms is strongly associated with increased risk of negative work-related outcomes. For example, disrupted sleep, hot flashes, fatigue, and emotional fluctuations can diminish job satisfaction and psychological well-being [15]. Women in the top quartile for menopausal symptom severity are 15.6 times more likely to experience adverse effects at work than those experiencing lower symptoms [13]. To date, symptom-level menopausal experiences remain underexplored, yet understanding their impact on work quality is crucial for fostering inclusive workplaces.

Workplace social support is widely recognised as a protective factor that can enhance employee mental health [17]. For menopausal women, support from colleagues and supervisors may reduce the psychological burden of symptoms and enhance confidence, disclosure, and job performance [18]. Conversely, lack of support can intensify distress, particularly when symptoms are stigmatised or misunderstood [18]. Indeed, workplace support, including increased awareness of menopause, flexible working hours, and strong supervisor support, has been shown to reduce the impact of menopause symptoms at work [15]. A recent qualitative study in the UK police force found that stigmatising menopause cultures at work deter disclosure and help-seeking, entrench stigma, and pressure women to hide symptoms to fit “ideal worker” norms [19].

Menopause symptom severity may also affect outcomes such as absenteeism and reduced working hours. A 2023 survey reported that 34 % of women had taken time off due to menopause symptoms, with 18 % absent for more than three days [15]. Symptoms most associated with absenteeism include somatic and psychological symptoms like fatigue, hot flushes, mood disturbances. However, women may avoid disclosing the reason for absence due to stigma, leading to underreporting [20]. In addition to absenteeism, presenteeism is common and may further degrade productivity. Studies on related health issues, like dysmenorrhoea, demonstrate that symptom severity, low self-efficacy, and unsupportive environments contribute to both absenteeism and presenteeism, creating a cycle of reduced well-being and job performance [21]. Such patterns may also apply to menopause, though research in this area remains sparse.

While Ireland recognises menopause as a health and occupational issue, workplace-focused research remains limited. As such, this preliminary study examines how menopause symptom severity relates to occupational self-efficacy, work ability, perceived health, support, and absenteeism, aiming to motivate larger population-wide studies that inform practices sustaining midlife careers and protecting mental health.

Based on the discussed literature, we predict the following hypotheses in Table 1:

**Table 1:** Summary of study hypotheses examining the impact of menopause symptoms on occupational outcomes.

Hypothesis	Description
H1	Psychological, urogenital, and somatic menopause symptoms will be significantly associated with occupational self-efficacy and work ability.
H2	There will be a relationship between menopause symptoms and health and work quality.
H3	Menopause symptom severity will be significantly associated with perceived workplace support.
H4	Menopause symptom severity will be associated with workplace absenteeism and fewer hours worked per week.

## 2 Methods

### 2.1 Participants

The sample included 123 respondents (M = 49.9 years, range = 37–62). See Table 2 for the demographic characteristics of participants. The inclusion criteria for this research required participants to be individuals over the age of 18, experiencing symptoms of perimenopause or menopause, and living and working in Ireland. Ethical approval for the study was granted by the Human Research Ethics Committee at Dublin Business School, in accordance with the Declaration of Helsinki. All participants provided informed consent prior to participation.

**Table 2:** Demographic characteristics of participants.

Characteristic	*n*	%
**Gender**		
Female	122	99.2 %
Non-binary	1	0.8 %
**Symptom Type∗**		
Post menopause Symptoms	51	41.5 %
Perimenopause Symptoms	72	58.5 %
**Current Treatment**		
Receiving Treatment	70	56.9 %
Not Receiving Treatment	49	39.8 %
Waiting for Treatment	4	3.3 %
**Hours worked per week**		
**15**–**20**	17	13.8 %
**20**–**35**	29	23.6 %
**35**–**40**	55	44.7 %
**40+**	20	16.3 %

*Note.* N = 123. Participants were, on average, 49.9 years old (SD = 4.92) (Range = 37–62). ∗ Participants self-identified the menopausal phase they believed they were currently experiencing.

### 2.2 Design

The current study is a quantitative, within-subjects, online survey using a correlational design. Participants were recruited through purposive and snowball sampling on online/social media platforms. Recruitment materials included a poster that identified the study as examining workplace experiences of menopause and outlined the key inclusion/exclusion criteria mentioned above. Participants were invited to complete the survey using Microsoft Forms. Before completing the survey, participants were provided with an information sheet and a consent form, followed by a debrief sheet upon completion of the survey.

### 2.3 Materials

The survey included demographic and inclusion items along with five standardised measures: the Menopause Rating Scale (MRS) [20], the Occupational Self-Efficacy Scale (short form) [22], the Work Ability Index (WAI) [23], the Health and Work Questionnaire [24], and the Perceived Workplace Support Scale [25].

#### 2.3.1 Menopause symptoms

Menopausal status was assessed via a single self-report item in which participants indicated whether they believed they were currently perimenopausal or postmenopausal. Menopause symptoms were assessed using the Menopause Rating Scale (MRS) [26], which was developed to evaluate the severity of menopause symptoms and their impact on quality of life. The MRS is an 11-item self-report questionnaire comprising three subscales designed to measure somatic, psychological, and urogenital symptoms. The somatic subscale evaluates physical symptoms, with questions such as, “Have you experienced hot flushes or night sweats?” to capture vasomotor disturbances. The psychological subscale addresses emotional and cognitive changes, including questions like, “Have you felt down, sad, or lacking in motivation?” to assess mood and affect. Lastly, the urogenital subscale focuses on genitourinary health and sexual function, asking participants, for example, “Have you experienced vaginal dryness or discomfort?”

Each item is rated on a 5-point Likert scale based on symptom severity: 0 = No symptoms, 1 = Mild, 2 = Moderate, 3 = Severe and 4 = Very severe. Each dimension can be scored and reported separately, or the composite score can be reported. Total composite scores range from 0 to 44, with higher scores indicating more severe menopause symptoms. In the current study, internal consistency was assessed using Cronbach's Alpha, which indicated that the MRS demonstrated good reliability (α = .808, N = 11 items).

#### 2.3.2 Workplace Self-Efficacy

Workplace Self-Efficacy was measured using the Occupational Self-Efficacy Scale (OSE-SF) [22], a short-form scale designed to assess an individual's belief in their ability to perform work-related tasks and cope with occupational challenges successfully. The OSE-SF is a 6-item self-report questionnaire rated on a 6-point Likert scale ranging from 1 = strongly disagree to 6 = strongly agree. Each item assesses the individual's confidence in their ability to perform effectively at work, for example, “*Whatever comes my way in my job, I can usually handle it”*. In this study, the scale demonstrated excellent internal consistency (α = .916), consistent with prior research.

#### 2.3.3 Work ability

A Modified version of the Work Ability Index (WAI) [23] was used in this study, incorporating 2 of the seven original dimensions. Dimensions 1 and 2 focus on self-perceived work ability and its relationship to physical and mental job demands. Dimension 1 asks respondents to rate their current work ability on a scale of 0 (completely unable to work) to 10 (work ability at its lifetime best). Dimension 2 asks respondents to evaluate how well they can meet the physical and mental demands of their job, rated on a 5-point scale ranging from 1 (very poor) to 5 (very good). Scores are then converted into a 2–10 scale per the original WAI scoring instructions. Total scores range from 2 to 20, with higher scores representing better overall work ability. This modified version offers a simplified assessment of work ability, focusing on immediate self-perceived work ability rather than long-term health conditions, while retaining the core elements of the original scale. The full WAI has demonstrated good reliability (α = .80); for this study, internal consistency analysis of the modified version yielded a Cronbach's Alpha of 0.767, suggesting acceptable reliability.

#### 2.3.4 Health and work

The Health and Work Questionnaire (HWQ), developed by Shikiar et al. [24], is a multidimensional instrument designed to assess workplace productivity. The HWQ is a 24-item self-report questionnaire with six subscales: Productivity (Split into Efficacy, Quality, Quantity), Concentration/Focus, Supervisor relations, Non-work Satisfaction, Work Satisfaction and Impatience/Irritability. Participants are asked to respond to questions, for example, “*How much control did you feel you had over how you did your job this week?”* using a 10-point Likert scale with higher scores indicating a more favourable outcome in each dimension. Items from the Impatience/Irritability subscale were reverse-scored so higher scores indicate lower impatience/irritability; these items were recoded before analysis. Scores were calculated by averaging the responses within each subscale. In the present study, internal consistency for the HWQ total score was α = .93.

#### 2.3.5 Workplace support

Participants' perception of workplace support was assessed using the Perceived Workplace Support Scale (PWSS) [25]. The PWSS is a self-report instrument designed to measure employees’ perceptions of support within their workplace. The scale is made up of 19 items and is divided into three subscales: manager support (“*My manager understands my work-related needs*”), coworkers support (“*My co-workers are willing to help me when needed”*) and overall support provided by the organisation (“*My organisation values employee well-being*”). Each item is rated on a 5-point Likert scale from 0 = strongly disagree to 4 = strongly agree. Reverse scoring is used to ensure consistency when answering questions relating to adverse outcomes. The measure was scored in accordance with the instructions, including both scored and reverse-scored items. Higher scores indicate greater levels of perceived support, and subscale scores were analysed and reported separately, as recommended by the scale developers. In the current research, internal consistency for Manager Support was α = .86, Co-worker Support was α = .88, and Organisational Support was α = .92.

## 3 Data analysis

Descriptive statistics were computed for all variables included in the study in Table 3. More than half of the participants reported perimenopause symptoms (58.5 %), and 56.9 % were currently receiving treatment. Regarding MRS severity categorisation of participants: 41.3 % (N = 51) were classified as mild, 57.0 % (N = 70) as moderate, and 1.7 % (N = 2) as severe. A post-hoc power analysis was conducted using G∗Power to assess the adequacy of the largest regression model (N = 123, 3 predictors). With an observed R^2^ = 0.177 (f^2^ = 0.215) and α = .05, the analysis indicated a power of 0.99**,** suggesting the model was well-powered to detect medium-sized effects.

**Table 3:** Means and standard deviations (SDs) for all scales included in the study.

Scale	*M*	*SD*
MRS Total	16.38	6.43
MRS: Psychological	7.05	3.11
MRS: Somatic	5.47	2.44
MRS: Urogenital	3.81	2.35
HWQ Total	6.91	1.30
HWQ: Work Satisfaction	6.42	1.81
HWQ: Non-work Satisfaction	6.90	1.89
HWQ: Supervisor Relations	6.94	2.24
HWQ: Productivity	7.34	1.41
HWQ: Impatience/Irritability	7.12	1.92
HWQ: Concentration/Focus	5.48	1.49
HWQ: Productivity (Efficacy)	7.38	1.53
HWQ: Productivity (Quality)	7.70	1.47
HWQ: Productivity (Amount)	7.61	1.69
Work Ability Index	21.89	4.24
Occupational Self-Efficacy Scale	28.27	5.26
Perceived Workplace Support – Co-Workers	22.57	6.07
Perceived Workplace Support – Manager	10.56	4.05
Perceived Workplace Support – Organisation	16.87	6.37

For regressions and correlations, assumptions of linearity, normality, homoscedasticity, and multicollinearity were assessed. All assumptions were adequately met. A threshold of p > .05 was established to consider significance. Statistical analyses were conducted using JASP [27].

## 4 Results

### 4.1 Hypothesis 1. Psychological, urogenital, and somatic menopause symptoms will be significantly associated with occupational self-efficacy and work ability

Multiple linear regressions were conducted to examine whether somatic, psychological, and urogenital menopause symptoms were associated with occupational self-efficacy and work ability.

The model for occupational self-efficacy was significant (R^2^ = 0.18, F(3, 119) = 8.52, p < .001), indicating that approximately 18 % of the variance in occupational self-efficacy scores was explained by the three symptom domains. Among the predictors, psychological symptoms significantly negatively predicted occupational self-efficacy (β = −0.53, t(119) = −4.32, p < .001). However somatic symptoms (β = 0.12, t = 1.08, p = .282) and urogenital symptoms (β = 0.10, t (119) = 1.06, p = .289) were not statistically significant predictors. See Table 4 for full reported regression results.

**Table 4:** Regression coefficients for the association of somatic, psychological, and urogenital menopause symptoms with occupational self-efficacy.

Predictor	b	SE	β	t	p	95 % Confidence Intervals
(Intercept)	32.343	1.230		26.292	<0.001	29.906, 34.779
MRS: Somatic Symptoms	0.269	0.248	0.123	1.081	0.282	−0.223, 0.761
MRS: Psychological Symptoms	−0.910	0.211	−0.530	−4.318	<0.001	−1.328, −0.493
MRS: Urogenital Symptoms	0.228	0.214	0.102	1.064	0.289	−0.196, 0.653

Note. b = unstandardised coefficient; SE = standard error; β = standardised coefficient. p-values are two-tailed.

The model for work ability was also significant, R^2^ = 0.22, F(3, 116) = 10.77, p < .001. This indicates that approximately 22 % of the variance in work ability scores was explained by the symptom domains. Among the predictors, psychological symptoms significantly predicted lower work ability (β = −0.43, t (119) = −3.65, p < .001). Somatic symptoms (β = 0.02, t (119) = 0.16, p = .876) and urogenital symptoms (β = −0.09, t (119) = −0.93, p = .352) were not statistically significant predictors. See Table 5 for full reported regression results.

**Table 5:** Regression coefficients for the association of somatic, psychological, and urogenital menopause symptoms with work ability.

Predictor	b	SE	β	t	p	95 % Confidence Intervals
(Intercept)	26.538	0.966		27.471	<0.001	24.624, 28.451
MRS: Somatic Symptoms	0.030	0.189	0.017	0.157	0.876	−0.345, 0.404
MRS: Psychological Symptoms	−0.600	0.164	−0.430	−3.646	<0.001	−0.925, −0.274
MRS: Urogenital Symptoms	−0.156	0.167	−0.087	−0.934	0.352	−0.486, 0.175

Note. b = unstandardised coefficient; SE = standard error; β = standardised coefficient. p-values are two-tailed.

Taken together, these findings suggest that greater psychological menopause symptoms are associated with lower occupational self-efficacy and work ability, while somatic and urogenital symptoms do not significantly relate to either.

### 4.2 Hypothesis 2. There will be a relationship between menopause symptoms and health and work quality

**Fig. 1:**
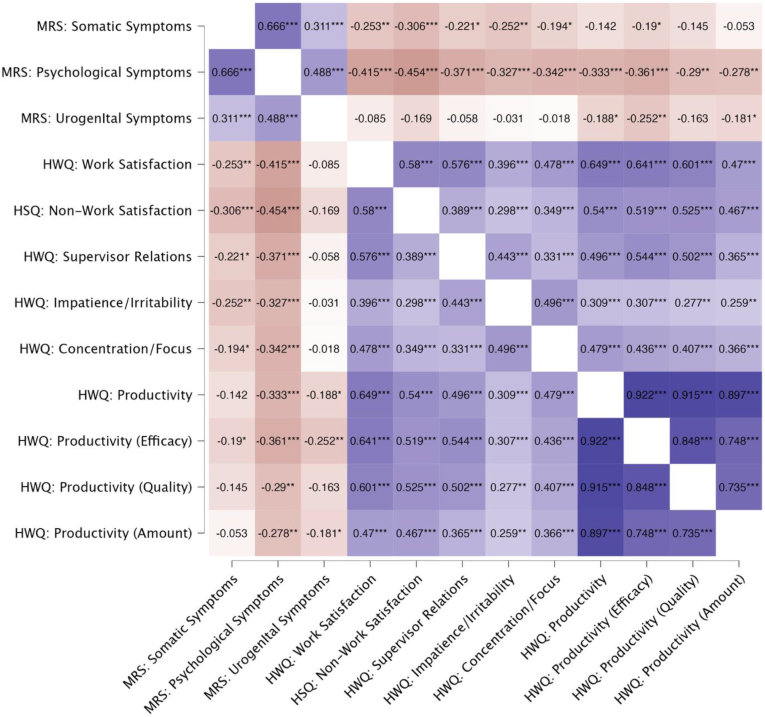
Pearson's R correlations between menopause symptoms and health and work quality subscales. Statistical significance as follows: ∗p < .05, ∗∗p < .01, ∗∗∗p < .001. Purple boxes signify positive correlations, brown boxes signify negative correlations.

Pearson's R correlation analyses were conducted to examine the relationships between menopause symptoms and subscales from the health and work quality questionnaire (See Fig. 1. For full reported Pearsons R correlations between all variables),

The MRS psychological symptoms showed consistent negative associations with all HWQ domains. Higher psychological symptoms were related to lower work satisfaction (*r* = −0.49, *p* < .001), non-work satisfaction (r = - 0.42, *p* < .001), poorer supervisor relations, (*r* = −0.37, *p* < .001), reduced concentration and focus, (*r* = −0.34, *p* < .001), and higher workplace impatience and irritability (*r* = −0.33, *p* < .001). The MRS psychological symptoms were also related to lower overall productivity (*r* = −0.33, *p* < .001), and its subcomponents: (efficacy, *r* = −0.36, *p* < .001); (quality, *r* = −0.29, *p* = .001); and amount (*r* = −0.28, *p* = .002).

The MRS somatic symptoms were significantly negatively correlated with lower work satisfaction (*r* = −0.25, *p* = .005), non-work satisfaction (r = −0.31. *p* < .001) poorer supervisor relations (*r* = −0.22, *p* = .014), greater impatience and irritability (*r* = −0.25, *p* = .005), reduced concentration and focus (*r* = −0.19, *p* = .032) and reduced productivity efficacy (*r* = −0.19, *p* = .037). Other aspects of productivity, including overall productivity, quality, and amount, were not significantly associated with somatic symptoms.

MRS Urogenital symptoms were associated with reduced overall productivity (*r* = −0.19, *p* = .038), productivity efficacy (*r* = −0.25, *p* = .005), and productivity amount (*r* = −0.18, *p* = .046). No significant relationships were found between urogenital symptoms and work satisfaction, supervisor relations, impatience/irritability, concentration/focus, or productivity quality.

### 4.3 Hypothesis 3: Menopause symptom severity will be significantly associated with perceived workplace support

A multiple linear regression was conducted to examine whether perceived support from managers, coworkers, and the organisation was associated with total menopause symptom severity (addition of the Psychological, Somatic and Urogenital subscales). The overall model was statistically significant, *R*^*2*^ = 0.77, *F*(3, 114) = 3.16, *p* = .028, indicating that 7.7 % of the variance in total symptom scores was explained by the model. Among the predictors, perceived organisational support was a significant negative predictor of symptom severity (β = −0.28, *t(114)* = −2.42, *p* = .017), suggesting that lower organisational support was associated with higher reported menopause symptoms. Support from managers (*B* = 0.24, *p* = .207) and coworkers (*B* = −0.13, *p* = .197) were not statistically significant predictors. See Table 6 for full reported regression results.

**Table 6:** Regression coefficients for the association of PWSS domains with total menopause symptom severity.

Predictor	b	SE	β	t	p	95 % Confidence Intervals
(Intercept)	21.517	2.445		8.800	<0.001	16.673, 26.361
PWSS Manager	0.235	0.185	0.146	1.270	0.207	−0.132, 0.602
PWSS Co-worker	−0.129	0.099	−0.123	−1.298	0.197	−0.325, 0.068
PWSS Organisation	−0.280	0.116	−0.282	−2.419	0.017	−0.509, −0.051

Note. b = unstandardised coefficient; SE = standard error; β = standardised coefficient. p-values are two-tailed.

### 4.4 Hypothesis 4: Menopause symptom severity will be associated with workplace absenteeism and fewer hours worked per week

No significant associations were found between somatic (*r* = −0.05, *p* = .578), psychological (*r* = −0.05, *p* = .580) and urogenital subscales (*r* = 0.10, *p* = .294) and the number of hours worked per week. Similarly, no significant associations were found between symptom domains and work absence amongst the somatic (*r* = −0.02, *p* = .790), psychological (*r* = 0.12, *p* = .198), and urogenital subscales (*r* = 0.04, *p* = .690). This indicates that menopause symptom severity is unrelated to self-reported workplace absenteeism and reduced hours worked per week.

### 4.5 Sensitivity analyses

#### 4.5.1 Is there a relationship between age and menopause symptom severity?

To examine whether symptom severity varies with menopausal progression, we treated age as a pragmatic proxy for stage within postmenopause and modelled age as a function of the three MRS domains (somatic, psychological, urogenital). The model was significant, *F*(3, 117) = 3.30, *p* = .023, explaining 8 % of the variance. Higher urogenital symptom severity was significantly associated with older age (β = 0.31, *t*(117) = 3.11, *p* = .002), whereas psychological (β = −0.21, *t*(117) = −1.60, *p* = .112) and somatic (β = 0.04, *t*(117) = 0.37, *p* = .711) symptoms were not (See Supplementary Material S1 for regression table)

#### 4.5.2 Is there a relationship between hours worked per week and menopause symptom severity?

We also assessed whether hours worked per week related to menopause symptom severity. Bivariate correlations between self-reported weekly hours worked and domain-specific symptom severity were non-significant (See Supplementary Material S2 for full reported correlation table). Accordingly, hours worked (and by implication, full-time vs. part-time status) was not retained as a covariate in the primary analyses conducted.

#### 4.5.3 Is there a difference in menopause symptom severity in those on treatment versus those not on treatment?

We conducted three one-way ANOVAs comparing menopause symptom severity between participants on treatment (N = 70) and not on treatment (N = 49) across the MRS domains. No group differences were detected in somatic symptom severity (*F*(1, 116) = 0.03, *p* = .870, ηp^2^ < 0.001); psychological symptom severity (*F*(1, 116) = 2.23, *p* = .138, ηp^2^ = 0.019); or urogenital symptom severity (*F*(1, 117) = 2.88, *p* = .093, ηp^2^ = 0.024). This indicates that there were no significant differences between symptom severity across the MRS domains in those on treatment compared to those not on treatment. Descriptive statistics (Means and SDs) are provided in Supplementary Material S3.

## 5 Discussion

This study examined the impact of menopause symptoms on occupational functioning and well-being, in working Irish women. Consistent with our first hypothesis, psychological menopausal symptoms were most strongly and consistently associated with occupational outcomes, including lower self-efficacy and diminished work ability. Large-scale population studies estimate that cognitive difficulties, such as problems with concentration and memory, affect up to 62 % of women during menopause [28]. Psychological and cognitive symptoms experienced during menopause are thought to stem from oestrogen deficiency, as oestrogen plays a neuroprotective role in supporting brain health and cognitive functioning. Declining oestrogen levels during the menopausal transition have been associated with reduced attention, slower information processing, and memory lapses [29]. Indeed, in the workplace, menopausal women often report challenges such as difficulty concentrating, delayed thinking, and forgetfulness when completing tasks [30]. These cognitive symptoms may relate to reduced self-reported work ability and lower occupational self-efficacy in our study. Interestingly, research indicates that these cognitive issues appear to be most pronounced during the perimenopausal stage, rather at the pre menopausal or post menopausal stages, highlighting this period as one of heightened vulnerability [31]. Given that a substantial portion of our sample consisted of women in perimenopause, these findings further support the view that this stage is particularly challenging for cognitive functioning and performance in the workplace.

In contrast, somatic and urogenital symptoms, while known to be physically distressing, were not significantly associated withwork ability and occupational self-efficacy. This aligns with findings from a study of 407 Irish hospital workers experiencing menopause, who reported that cognitive symptoms, such as fatigue, poor concentration, and memory difficulties, had the greatest impact on their job performance, as opposed to more somatic symptoms like hot flushes and night sweats [32]. However, the association between somatic/urogenital symptoms and work ability is likely moderated by job demands, with larger effects in shift-based and physically demanding occupations than in desk work. Without occupational detail, we are unable to assess heterogeneity in effects across job types. Additionally, somatic and urogenital symptoms prompt more treatment-seeking; as such subsequent symptom improvement may improve work outcomes. [50]. Notably, symptom severity was comparable among participants on treatment and those not on treatment. This may reflect domain-specific treatment efficacy, for example, hormone therapy effectively targets vasomotor symptoms but is less reliable for psychological or urogenital symptoms [51]. Capturing treatment type, dose, and duration in future research will allow clearer modelling of domain-specific improvements and their relevance for workplace outcomes.

In relation to our second hypothesis, greater severity of psychological menopausal symptoms was significantly associated with poorer outcomes across several health- and work-related domains. Specifically, higher symptom severity was associated with lower life and work satisfaction, higher irritability/impatience, poorer relationships with supervisors, reduced concentration and focus, as well as decreased perceived productivity across efficacy, quality, and quantity dimensions. This is consistent with prior research in the USA, showing that psychological symptoms such as low mood, anxiety, irritability, and cognitive difficulties are the most disruptive to performance, confidence, and motivation in work contexts [13]. In a study of Polish peri- and postmenopausal women employed in intellectual professions, significant associations were found between work-related stressors and various aspects of cognitive function—including memory, reaction time, attention, and processing speed—when completing work tasks. These findings highlight the considerable impact of psychological symptoms on perceived workplace productivity [33]. Furthermore, a recent study by Hobson and Dennis [34] highlights that women experiencing more severe psychological menopause symptoms often report an inability to enjoy life, citing the strain of balancing work, fatigue, and family responsibilities. Other studies support the idea that psychological distress during menopause is often intensified by the workplace and supervisors. For instance, Hardy et al. [35] examined female employees’ perspectives on their employers and supervisors, highlighting how certain aspects of the work environment, such as limited awareness or understanding among colleagues, poor communication skills among staff and management, and the absence of organisational policies, contributed to their difficulties in managing menopause symptoms at work.

We also found that somatic symptoms were associated with reduced life and work satisfaction, poorer supervisor relations, greater impatience and irritability and reduced productivity efficacy. Indeed, research suggests that somatic symptoms, such as hot flashes, pose significant challenges in the workplace, as they can undermine self-confidence and impair short-term memory and concentration [36]. In addition, other somatic symptoms such as sleep disturbances, may lead to chronic fatigue and irritability, further exacerbating cognitive difficulties and contributing to reduced work productivity and efficiency [37]. We also found that urogenital symptoms only showed associations with reduced work output and efficiency, suggesting their impact in the workplace may be limited. Indeed, whilst some research indicates that urogenital symptoms primarily affect sexual health and have minimal influence on daily functioning [38]. Other research indicates that urogenital symptoms may be more burdensome in physically demanding jobs [52]. The absence of occupation/industry data prevents us from assessing whether work context modified the observed symptom associations.

These findings further support our third hypothesis, which showed that greater perceived organisational support was significantly associated with lower overall menopausal symptom severity, whereas support from individual managers or coworkers alone had no significant effect. This is consistent with the existing literature, which suggests that certain workplace factors can either intensify or reduce menopausal symptoms. Indeed, limited awareness and understanding of menopause, poor communication among staff and leadership, and the lack of formal organisational menopause policies have been identified as contributing factors [39]. Although menopausal symptoms can significantly affect women's work lives, open dialogue about these experiences is often avoided. In an Australian study of 1092 female employees, most reported feeling uneasy about raising the topic with their direct supervisors due to fears of being dismissed, mocked, or excluded by both peers and management [6]. This underscores the importance of formal structures and visible workplace policies in fostering open dialogue about menopause and shaping women's experiences in professional settings. Additionally, the presence of an organisational culture that recognises menopause as a legitimate workplace issue may provide psychological reassurance and reduce internalised stigma [40]. In contrast, the absence of formal recognition and top-down organisational support may in turn compel women to conceal symptoms or refrain from requesting accommodations, increasing emotional strain and exacerbating symptoms [41].

Interestingly, contrary to our fourth hypothesis, the severity of menopausal symptoms was not associated with self-reported absenteeism or reduced working hours, despite the occupational impact of psychological symptoms. This may suggest that menopausal symptoms do not interfere with formal participation. Indeed, across studies, an estimated 15–40 % of menopausal women report difficulty managing symptoms at work [30,53,54]. While considerable, this also means most do not report such difficulties and menopause symptoms may not necessarily interfere with formal participation. This could alternatively highlight a hidden burden as women may feel compelled to maintain full attendance due to workplace expectations, fear of stigma, or lack of formal recognition of their symptoms. This mirrors patterns in menstruation research, where individuals report working through pain or distress without taking time off or seeking support [42]. This hidden burden may contribute to presenteeism, where employees are physically present but experience reduced productivity, increased fatigue, or emotional exhaustion. While not captured through absenteeism statistics, presenteeism represents a significant cost to both employee well-being and organisational effectiveness. Future research should integrate measures of presenteeism and productivity loss better to understand the full impact of menopause on working life.

## 6 Implications and applications

The findings have important implications for workplace policy and practice. As menopause increasingly enters public discourse in Ireland, employers must transition from awareness to action. Our preliminary findings underscore the need to prioritise mental health and employee wellbeing during menopause to ameliorate its psychological impacts. In 2023, the Irish government introduced formal guidelines for managing menopause in the public sector, which included training, awareness campaigns, and recommendations for flexible working [43]. However, implementation remains inconsistent and its efficacy thus far has not been evaluated. Without accountability structures, these guidelines risk becoming symbolic rather than transformative. Given the links between psychological symptoms and work outcomes, future research should pilot and evaluate workplace interventions. Existing evidence suggests that self-guided CBT can enhance mental health at work, reduce presenteeism, and improve work and social functioning, while menopause-awareness programmes increase knowledge and foster more positive attitudes among employees and line managers/supervisors [44,59]. Another study (n = 896) found that flexible working, managerial awareness, access to information, temperature regulation, and informal support mechanisms were beneficial to women experiencing menopause [30]. To date, Ireland lacks population-wide investigations of menopause in the workplace; as such, the results of this preliminary study should motivate adequately powered, longitudinal and mixed-methods research to replicate and extend these findings and to provide a basis for policy and practice. Finally, addressing the ongoing stigma around menopause is essential for organisations in improving psychological wellbeing for employees. In 2021, the European Menopause and Andropause Society released evidence-based guidance for women, employers, and healthcare professionals, emphasising the importance of increasing menopause awareness, reducing stigma, offering flexible work options, and improving workplace conditions [45]. Extending menopause education to schools and community settings as part of public health initiatives could also help foster a more inclusive and informed societal approach to menopause.

## 7 Limitations and future directions

To our knowledge, this is the first study in the Republic of Ireland to examine such a broad set of self-reported work outcomes among women experiencing menopause. However, the findings of this study are preliminary, and should be interpreted in light of several limitations. The reliance on self-report work performance measures, including self-reported absenteeism and reductions in work hours, may introduce response bias. Participants may underreport these outcomes due to social desirability or discomfort in disclosing work-related impairments, particularly in the context of menopause. Furthermore, self-perceived work outcomes may diverge from objective work performance indicators (e.g., appraisals, productivity metrics); nevertheless, one's perceived capacity and ability remain important to assess because they shape day-to-day engagement, self-regulation, help-seeking, and intentions to remain in or exit a role [55]. Moreover, two of the MRS subscales (Somatic and Urogenital) demonstrated medium reliability, which may reduce the precision of certain relationships and associations in the study. Additionally, menopausal trajectories are heterogeneous, and stage definitions based solely on self-report are contested [56]. In our sample, urogenital symptoms were higher in older participants, consistent with evidence that these symptoms are more pronounced in later postmenopause [57]. However, age is an imperfect proxy and we did not capture the timing of the final menstrual period to confirm the postmenopause stage. Future work should collect granular staging data (e.g., final menstrual period; early vs. late postmenopause) and model heterogeneity to clarify links between symptom severity and workplace outcomes.

Additionally, we did not measure several established confounders of self-reported workplace wellbeing beyond menopause symptoms (e.g., job role/sector, workload, tenure in role, education, income, work environment and parity [58]. Given that workplaces and employers are heterogeneous; Future studies should capture and adjust for these variables to estimate effects independent of menopause symptom severity. Comparative studies involving non-menopausal or asymptomatic women, as well as men, could further isolate menopause-specific effects. While quantitative data provide clarity on general patterns - qualitative insights, or incorporating patient and public involvement, can illuminate how women make sense of their experiences, cope with symptoms, and navigate disclosure in the workplace. Finally, exploring intersectional factors, including age, ethnicity, and socioeconomic status, would also strengthen the understanding of how menopause is experienced across different population groups.

## 8 Conclusion

The results of this preliminary study show that psychological menopause symptoms are associated with lower occupational self-efficacy, reduced work ability, poorer perceived health and work quality among women in Ireland. We also identify organisational support as a key factor associated with symptom severity. Notably, somatic and urogenital symptoms were not significantly associated with work ability and self-efficacy, suggesting that the evidence for the effect of menopause symptoms on workplace outcomes may be mixed and larger studies are needed. Employee supports should be designed to promote inclusion while explicitly guarding against the reproduction of stigma or inequality. As the literature cautions, menopause-focused initiatives, and research of menopause in the workplace, must not become instruments of workplace prejudice or a pretext for discriminatory treatment of women [41]. Evidence shows that workers of all ages, genders, and health statuses fare better when treated as individuals, offered choice and flexibility, and are listened to and supported [52]. By embedding menopause awareness into organisational culture, providing education and resources, and pairing policy with meaningful action, workplaces can reduce stigma and barriers for midlife women and improve the experience of menopause at work.

## CRediT authorship contribution statement

**Wendyrose Smith:** Writing – original draft, Validation, Software, Resources, Methodology, Investigation, Formal analysis, Data curation, Conceptualization. **Sarah M. Cooney:** Writing – review & editing, Validation, Conceptualization. **Akansha M. Naraindas:** Writing – review & editing, Writing – original draft, Visualization, Validation, Supervision, Resources, Project administration, Formal analysis, Conceptualization.

## Ethics approval

The study was conducted in line with the Declaration of Helsinki and approved by the Institutional Review Board of Dublin Business School (code PY2425-129 approved 20/12/2024).

## Availability of data

The data are available upon request from the corresponding author.

## Funding

This project was funded by the Dublin Business School Research Grant (Project code: 2025_6) awarded to Dr Akansha M. Naraindas.

## Declaration of competing interest

The authors declare that they have no known competing financial interests or personal relationships that could have appeared to influence the work reported in this paper.
