# Supplementary material for: The influence of menopause symptoms on workplace mental health among Irish women: A preliminary study
Source: Compr Psychoneuroendocrinol. 2025 Oct 22;24:100324. doi: 10.1016/j.cpnec.2025.100324 (PMC12595140; doi:10.1016/j.cpnec.2025.100324)
Supplement: Multimedia component 1 [file mmc1.docx]

**Supplementary Material**

**Supplementary material S1:** Regression coefficients for menopause symptom domains predicting age

| Predictor | b | SE | β | t | p |
| --- | --- | --- | --- | --- | --- |
| (Intercept) | 49.183 | 1.199 |  | 41.014 | < .001 |
| MRS: Somatic Symptoms | 0.089 | 0.239 | 0.044 | 0.371 | .711 |
| MRS: Psychological Symptoms | -0.328 | 0.205 | -0.206 | -1.599 | .112 |
| MRS: Urogenital Symptoms | 0.658 | 0.211 | 0.314 | 3.114 | .002 |

Note. b = unstandardised coefficient; SE = standard error; β = standardised coefficient. p-values are two-tailed.

**Supplementary material S2:** Correlation table between hours worked per week and menopause symptom severity (Values are Pearson's r; two-tailed p-values in parentheses)

| **Variable** | **MRS: Somatic Symptoms** | **MRS: Psychological Symptoms** | **MRS: Urogenital Symptoms** | **Hours worked per week** |
| --- | --- | --- | --- | --- |
| 1. MRS: Somatic Symptoms | — |  |  |  |
| 2. MRS: Psychological Symptoms | 0.666 (< .001) | — |  |  |
| 3. MRS: Urogenital Symptoms | 0.311 (< .001) | 0.488 (< .001) | — |  |
| 4. Hours worked per week | -0.051 (.578) | -0.051 (.580) | 0.095 (.294) | — |

**Supplementary Material S3.** Means and Standard deviations (in brackets) in menopause symptom severity between those on treatment and those not on treatment.

| **Treatment status** | **MRS Somatic** | **MRS Psychological** | **MRS Urogenital** |
| --- | --- | --- | --- |
| Not on treatment | 5.46 (2.62) | 7.40 (3.18) | 4.11 (2.39) |
| On treatment | 5.39 (2.27) | 6.52 (3.09) | 3.37 (2.33) |
